# Pedestrian injuries in collisions with pedal cycles in the context of increased active travel: Trends in England, 2005–2015

**DOI:** 10.1016/j.jth.2022.101340

**Published:** 2022-03

**Authors:** Tika Ram, Judith Green, Rebecca Steinbach, Phil Edwards

**Affiliations:** a Faculty of Public Health and Policy, London School of Hygiene & Tropical Medicine, London, UK; b National Infection Service, UK Health Security Agency, 61 Colindale Ave, NW9 5EQ, London, UK; c Wellcome Centre for Cultures & Environments of Health, University of Exeter, Exeter, UK; d National Centre for Social Research, London, UK; e Faculty of Epidemiology and Population Health, London School of Hygiene & Tropical Medicine, London, UK

**Keywords:** Active travel, Mode shift, Road traffic injuries, Pedestrians, Cyclists

## Abstract

**Introduction:**

Increasing levels of active travel in the population brings many public health benefits, but may also change the risks of road injury for different road users. We examined changes in rates of pedestrian injuries resulting from collisions with pedal cycles and motor vehicles in England during 2005–2015, a period of increased cycling activity, and described the gender, age distribution and locations of pedestrians injured in collisions with pedal cycles and motor vehicles.

**Methods:**

Collisions data were obtained from police STATS19 datasets**.** We used two measures of cycle/motor vehicle use; miles per annum, and estimated average travel time, and assessed evidence for trends towards increase over time using Poisson regression analysis.

**Results:**

There were 3414 pedestrians injured in collisions with one or more pedal cycles in England during 2005–2015, 763 of whom were killed or seriously injured (KSI). This accounted for 1.3% of the total pedestrians KSI from all vehicles. Of those KSI in collisions with cycles, 62% were female; 42% over the age of 60; 26% were on the footway or verge and 24% were on a pedestrian crossing. There was a 6% (IRR 1.056; 95% CI 1.032–1.080, p < 0.001) annual increase in the pedestrian KSI rate per billion vehicle miles cycled in England over the time span. This increase was disproportionate to the increase in cycle use measured by vehicle miles or time spent cycling.

**Conclusions:**

Increases in cycling were associated with disproportionate increases in pedestrian injuries in collisions with pedal cycles in England, although these collisions remain a very small proportion of all road injury. Increased active travel is essential for meeting a range of public health goals, but needs to be planned for with consideration for potential impact on pedestrians, particularly older citizens.

## Highlights

• Pedestrian injuries from collisions with cycles increased between 2005 and 2015.
• They remain a small proportion of all pedestrian injuries.
• The increase was disproportionate to the increase in miles and hours cycled.
• Older pedestrians appeared to be at relatively higher risk.
• Cycling infrastructure must protect both cyclists and pedestrians.

## 1 Introduction

Shifting transport mode use towards active modes (predominantly walking and cycling) brings gains for population health, through increased physical activity, reductions in pollution, and potential co-benefits such as improved neighbourhood cohesion (De Nazelle et al., 2011; Saunders et al., 2013, Khan et al., 2020). Increased levels of walking and cycling also tend to reduce injury risks to both groups of road user over time, as a result of a ‘safety in numbers’ effect (Elvik and Goel 2019). Active travel policies are, then, in general, a ‘win-win’ for achieving multiple sustainability and public health goals, and between 1990 and 2015, across many European and North American cities, cycling rates increased around three or four-fold (Pucher and Buehler 2017). However, any shift in mode use is likely to change the relative risks for different road users, particularly where these have been brought about through road infrastructure changes that bring together new mixes of road user.

The impact on pedestrians from increased cycling rates has been relatively under-explored. The number of pedestrian casualties has been falling over the last three decades in most high-income countries (Buehler and Pucher, 2017), but they remain a significant contributor to the worldwide burden of disease (Khan et al., 2020). In the UK, for instance, there were 5588 pedestrians killed or seriously injured (KSI) in 2016, representing 22% of all people KSI on British roads that year (Department for Transport, 2017a). The majority of these pedestrian casualties occur in collisions involving cars and other motor vehicles (Department for Transport, 2017a). However, a small number of pedestrians are injured in collisions with other vehicles such as pedal cycles, ridden horses, trams and mobility scooters (Department for Transport, 2017a).

There is an extensive body of research on the epidemiology of collisions involving motor vehicles, to both pedestrians and cyclists, but little to date on pedestrian casualties from collisions with pedal cycles. In part, this is because they form a relatively minor proportion of road casualties (Grzebieta et al., 2011). However, increasing investments in new infrastructure to accommodate and encourage cycling in many cities across the world raise significant questions about the relative risks to different groups of road users (Chong et al., 2010; Haworth et al., 2014). To date, most studies and reviews of infrastructure have focused on cyclists' safety (Thomas and DeRobertis, 2013; Zangenehpour et al., 2016), or on pedestrians' perceptions of safety (Gkekas et al., 2020; Nikiforiadis and Basbas 2019). Reynolds et al. (2009), for instance, identified that purpose-built cycling tracks that separated pedal cycles from motorised traffic can reduce the risks to cyclists; although a more recent Cochrane review (Mulvaney et al., 2015) noted the lack of robust evidence to make any firm conclusions about which kinds of infrastructure do reduce risks to cyclists, given the challenges of comparing different infrastructure types and of controlling for changing exposure as a result of infrastructure implementation. Case cross-over studies in Canada (Teschke et al., 2012) and London (Aldred et al., 2018), comparing the place of a cyclist's injury to randomly chosen other places on their route to identify how infrastructure impacts on risk, address these weaknesses to some extent. Aldred et al. (2018) found that reduced traffic speeds and volumes, residential streets and cycling density all reduce the odds of cycling injuries. However, they did not examine impact on pedestrian injury.

The few studies to date that do address injury risks for pedestrians include Chong et al. (2010), who examined injuries in New South Wales, Australia, and identified concerns about shifting injury risks from cyclists to pedestrians when cyclists and pedestrians use the same roadways. This concern was echoed in an observational study of conflicts between pedestrians and cyclists in the USA, where Tuckel et al. (2014) suggested the observed decrease in incidence of pedestrian injuries from cyclists in California and New York states between 2004 and 2011 might be due to reduced exposure of children to cyclists as well as improvements in cycling infrastructure (Tuckel et al., 2014).

As urban administrations around the world aim to increase active travel, there is an urgent need to add to this limited body of evidence on the ways in which mode shift might change injury risks for pedestrians. This study therefore aimed to: identify the frequency of collisions and severity of pedestrian injuries from collisions with pedal cycles in England; estimate trends in rates of pedestrian injuries resulting from collisions with pedal cycles over the period 2005–2015; and to describe the age and sex profiles and locations of pedestrian casualties in collisions with pedal cycles. To put these data in context, we compare them with those describing pedestrian injuries from collisions with motor vehicles.

## 2 Materials and methods

### 2.1 Data sources

This cross-sectional analysis of road injury data investigated risks of fatal or serious pedestrian injury due to collision with a pedal cycle or a motorised vehicle in England from 2005 to 2015. Injury data for this study were obtained from the STATS19, police records of personal injury road collisions and resulting casualties that occur on the public highway in the UK (Department for Transport, 2016a). Not all pedestrian injuries are reported to the police. To minimize the effect of resulting reporting or recording bias, we limit injuries to those classified as killed or seriously injured (KSI) (Ward et al., 2006). Our analysis includes data up to 2015. After 2015, there were changes in the way injuries were categorised (Department for Transport, 2017b; 2018), particularly affecting the ways in which severity is recorded. A few police forces began adopting the new system earlier. A sensitivity analysis therefore examines KSI pedestrian injuries excluding police forces who adopted the system during our 2005–2015 study period.

Collisions resulting in injuries to pedestrians can be complex and involve multiple vehicles. To focus on trends in collisions involving pedestrians and pedal cycles, we exclude injuries to pedestrians sustained in a collision with cycles and other vehicles. We therefore examine all fatalities and serious injuries to pedestrians sustained in a collision with one or more pedal cycles. We compare these to pedestrian injuries sustained in a collision with one or more motor vehicles. To examine injury rates using travel survey-based measures of exposure (see below) we also examine pedestrian injuries involving one or more cars and vans. Sensitivity analyses examine fatal and serious pedestrian injuries sustained single-vehicle collisions (with pedal cycles and motor vehicles), all fatal and serious pedestrian injuries sustained in all collisions with pedal cycles (including those with motor vehicles), and fatal and serious pedestrian injuries sustained in all collisions with motor vehicles (including those with pedal cycles).

### 2.2 Measures of exposure

The major challenge for assessing relative injury risks by transport mode is that of identifying appropriate exposure measures, given that journeys by different mode are not comparable: they differ by average speed, length of journey and by the demographic profiles of those undertaking trips. Analyses using measures of distance travelled to characterise exposure may inappropriately compare the long journeys typically done by car with much shorter journeys by walking or cycling (Mindell et al., 2012). In the setting of our study (England), using time spent travelling may be preferable, given that the mean time spent on a cycling and driving trip is similar (Scholes et al., 2018) and that the overall time spent travelling tends to remain relatively constant across modes (Mindell et al., 2012). However, data on travel time by mode is reliant largely on self-report measures, which are subject to various biases (Tenenboim and Shiftan, 2016), including changes in the social desirability of mode choice over time. To offset the potential weaknesses of each measure, in this study we use two denominators to calculate rates, one generated from counts of vehicles (estimated annual vehicle traffic volume) and one from travel survey measures of time spent travelling by different modes.

Estimates of annual vehicle traffic volume come from counts of vehicles published in the Department for Transport's (DfT) Road Statistics (Department for Transport, 2017a). The DfT combines information from manual traffic counts with data from a network of automatic traffic counters to estimate daily traffic flow. Flow information is combined with data on road lengths to calculate number of vehicle miles travelled each year by vehicle type, reported in billion vehicle miles. As cyclists and pedestrians are not allowed on motorways in England, our analysis excludes vehicle miles on motorways.

Estimated average time spent travelling by mode is calculated by multiplying estimates from the National Travel Survey (NTS) on average hours travelled by person by year by mode of travel (Department for Transport, 2019a), by ONS mid-year population estimates. The NTS is a nationally representative survey of over 8000 households a year; within each selected household, all people aged over 5 years record in a travel diary the start, interchange (e.g. from bus to train) and end of every trip made in a seven-day period. Data collection takes place throughout the year, allowing for seasonal differences in travel.

A sensitivity analysis examines injury rates using estimated average distance travelled by mode. This is calculated by multiplying estimates from the NTS on average distance travelled per person by mode (Department for Transport, 2019a), by the mid-year population estimates available from the Office for National Statistics (ONS).

### 2.3 Analysis

We used Chi-squared tests to assess evidence for associations between gender and numbers KSI by vehicle type. Rates of pedestrian KSI were estimated using two measures of exposure: the first, traffic in billion vehicle miles, gives the KSI rate as ‘rate per billion vehicle miles'; the second measure of exposure, average time spend travelling, gives the KSI rate as ‘rate per million hours use’. Assuming a Poisson distribution for the number of casualties killed or seriously injured in time and space, approximate 95% confidence intervals (CI) were calculated for each rate. We used Poisson regression to estimate trends in the pedestrian KSI rate over the 11-year period using the two different exposure denominators. The KSI number was the dependent variable, year was independent variable, and exposure (denominator) was traffic volume (BVM) or average time spent travelling, respectively, for the two analyses. As a check for over-dispersion in the data, we also ran this analysis using a Negative Binomial regression model.

All data preparation and analyses were conducted using Stata (StataCorp 2017).

## 3 Results

### 3.1 Pedestrian injuries from collisions with pedal cycles

Between 2005 and 2015, there were 2,137,625 casualties from 1,780,653 reported road traffic accidents in England, 262,869 (12%) of which were pedestrians. Only 3414 (1.3%) of these pedestrian casualties were due to collisions with one or more pedal cycles with almost all those remaining 258,593 (98.4%) injured in collisions with one or more motor vehicles. In terms of traffic volume, pedal cycle traffic accounts for about 1% of all road traffic annually in England between 2005 and 2015. Of the 3414 pedestrian casualties from collisions with one or more pedal cycles, 763 (22.4%) had fatal or serious injuries. Of the 258,593 pedestrian casualties from collisions with one or more motor vehicles, 57,594 (22.3%) had fatal or serious injuries. (See Appendix, Table A1).

**Fig. 1:**
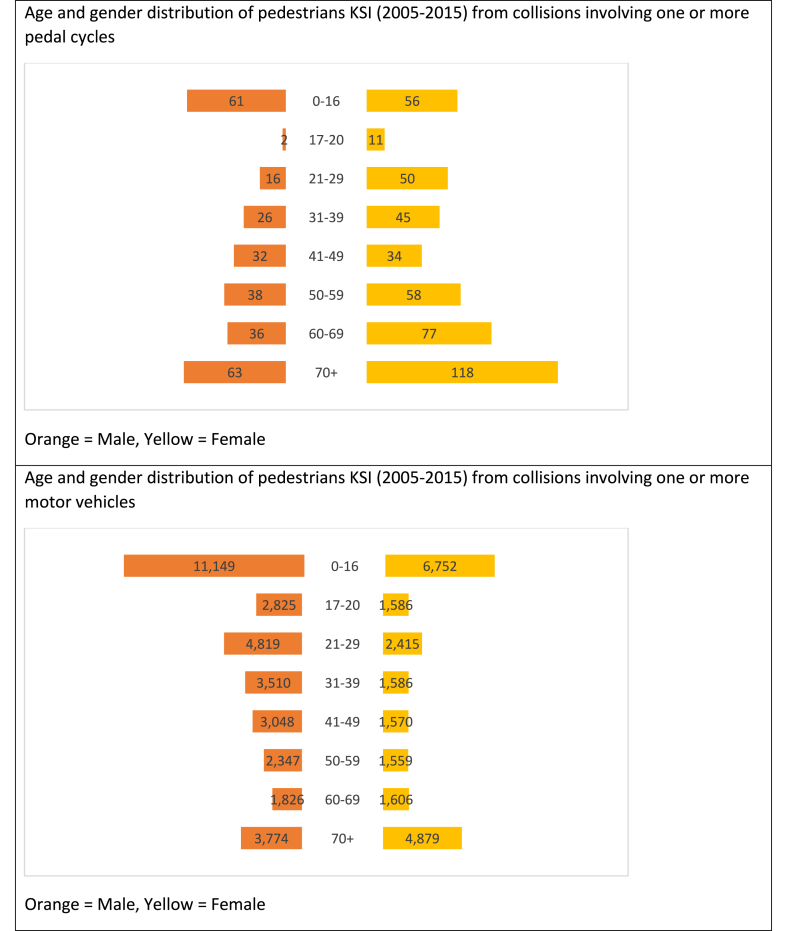
Age and gender distribution of pedestrians KSI.

The demographic characteristics of pedestrians injured in collisions with pedal cycles and motor vehicles differ: more females (62%) than males were KSI in collisions with cycles, whereas fewer females (40%) than males were KSI from collisions with motor vehicle. There was strong evidence of an association between gender and pedestrian KSI by vehicle type (p < 0.001) (Table 1). Pedestrian KSI casualties from pedal cycle collisions tended to be in older age groups, with a mean age of 50 (SD 24) and 42% over the age of 60, compared to those from motor vehicle collisions (mean age 36, SD 25; 22% over age 60) (Fig. 1).

**Table 1:** Gender distribution of KSI pedestrians by vehicle type.

Gender	Pedal Cycle (%)	Motor vehicle^a^ (%)	Chi-square test
			(p-value)
Male	289 (38)	34,787 (60)	
Female	474 (62)	22,799 (40)	<0.001
**Total**	**763**	**57,586**	

a 8 KSI pedestrians in motor vehicle crashes had missing gender information.

### 3.2 Trends over time

The overall percentage of pedestrians KSI in collisions with pedal cycles is no greater than 2% in any year (Table 2). During the period 2005–2015, the incidence of pedestrians KSI in collisions with motor vehicles started to decrease from 2006, whereas the incidence of KSI in collisions with pedal cycles increased, more than doubling since 2006. On average over the 11-year period, there were three pedestrian fatalities per year due to collisions with a pedal cycle and 67 seriously injured per year. For motor vehicle collisions during the same period, on average, there were 439 pedestrian fatalities per year and 4864 seriously injured casualties per year.

**Table 2:** Pedestrians KSI by year.

	Pedestrians KSI in collision involving one or more pedal cycle				Pedestrians KSI in collision involving one or more motor vehicle		
Year	Fatal	Serious	Total	% of total pedestrians KSI	Fatal	Serious	Total
2005	3	56	59	0.96%	568	5492	6060
2006	3	40	43	0.71%	589	5397	5986
2007	3	39	42	0.70%	549	5403	5952
2008	1	48	49	0.86%	490	5148	5638
2009	0	62	62	1.18%	433	4727	5160
2010	4	70	74	1.51%	337	4476	4813
2011	2	80	82	1.61%	381	4615	4996
2012	2	77	79	1.50%	348	4821	5169
2013	6	85	91	1.94%	327	4262	4589
2014	4	90	94	1.97%	372	4296	4668
2015	2	86	88	1.88%	342	4221	4563
**Total**	**30**	**733**	**763**	**1.30%**	**4736**	**52,858**	**57,594**

Pedal cycle traffic increased in England from 2005 until 2014 and then declined slightly. Overall, there was a 19% increase in pedal cycle traffic by 2015 compared to 2005. On average, there were 2.7 billion cycle miles per year in England over the study year period. The traffic volume for motor vehicles was more variable over time, with an overall 1% increase in motor vehicle traffic in 2015 compared to 2005. (See Appendix, Table A2).

### 3.3 Pedestrian locations

STATS19 data record pedestrian location at the time of collisions (Table 3). Of 763 pedestrians KSI in collisions with pedal cycles, 197 (26%) were on a footway or verge, 183 (24%) were crossing on a pedestrian crossing facility and 251 (33%) were crossing elsewhere on the road. In comparison, 8% of pedestrians KSI in collisions with motor vehicles were located on the footway or verge, 13% were on a crossing and 49% were crossing elsewhere on the road.

**Table 3:** Reported pedestrian locations at the time of collision (2005–2015).

Pedestrian's Location	Pedestrians KSI in collision with one or more pedal cycles		Pedestrians KSI in collision with one or more motor vehicles	
	Number	%	Number	%
Data missing	–	–	4	0.01
Crossing on pedestrian crossing facility	183	23.98	7736	13.43
Crossing in zig-zag approach lines	2	0.26	308	0.53
Crossing in zig-zag exit lines	0	0	280	0.49
Crossing elsewhere within 50m. of pedestrian crossing	45	5.9	5409	9.39
In carriageway, crossing elsewhere	251	32.9	28,467	49.43
On footway or verge	197	25.82	4442	7.71
On refuge, central island or central reservation	2	0.26	319	0.55
In centre of carriageway - not on refuge, island or central reservation	13	1.7	1889	3.28
In carriageway, not crossing	33	4.33	5333	9.26
Unknown or other	37	4.85	3407	5.92
**Total**	**763**	**100**	**57,594**	**100**

### 3.4 Rates of pedestrians KSI

**Fig. 2:**
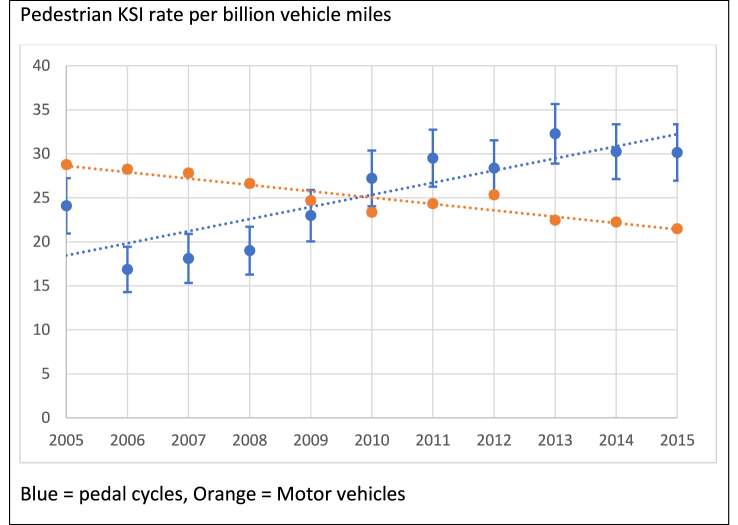
Pedestrian KSI rates from collisions with one or more pedal cycles compared to pedestrian KSI rates from collisions with one or more motor vehicles.

Fig. 2 shows the KSI rate over time for pedestrians in collisions involving one or more cycles and one or more motor vehicles, per billion vehicle miles. The rates of pedestrians KSI in collisions with a pedal cycle increased in England over the 11-year period, consistent with trends in the number of KSI from Table 1, while the rate of pedestrians KSI from collisions with motor vehicles declined.

**Fig. 3:**
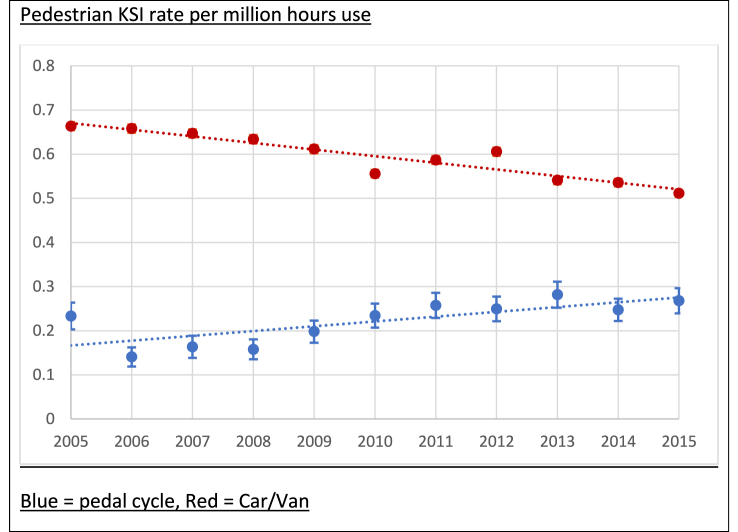
Pedestrian KSI rates from collisions with one or more pedal cycles compared to pedestrian KSI rates from collisions with one or more cars/vans.

Fig. 3 shows rates over time of pedestrians KSI in collisions involving one or more cycles and one or more cars or vans, per million hours use. Using this measure of exposure, rates of pedestrians KSI per million hours travel time from collisions involving one or more pedal cycles also appear to be increasing, and those involving one or more cars/vans decreasing.

On average over the 11-year period, there were 25.7 (95% CI: 23.9 to 27.5) pedestrians KSI per billion cycle miles per year in England, and 0.22 (95% CI: 0.21 to 0.24) pedestrians KSI per million hours cycled. In comparison, the average rate of pedestrians KSI from motor vehicle collisions was 25.18 (95% CI: 25.0 to 25.4) per billion vehicle miles and 0.59 (95% CI 0.59 to 0.60) pedestrians KSI per million hours travelled in a car or van.

Sensitivity analyses using average distance travelled as a denominator revealed similar trends over time to those using billion vehicle miles. Sensitivity analyses restricted to: (i) KSI pedestrian injuries sustained in single-vehicle collisions (with cyclists and motor vehicles); (ii) KSI pedestrian injuries sustained in all collisions with pedal cycles (including those with motor vehicles); and (iii) KSI pedestrian injuries sustained in all collisions with motor vehicles (including those with pedal cycles), identified identical trends to those KSI in collisions with one or more cycles.

### 3.5 Regression analysis

Overall, there was a 6% (IRR - 1.056; 95% CI: 1.032–1.080, p < 0.001) annual increase in the pedestrian KSI rate per billion vehicle miles in England 2005–2015 from collisions with pedal cycles, and a 3% annual reduction (IRR- 0.971, 95%CI: 0.969–0.974, p < 0.001) in pedestrians KSI from collisions with motor vehicles during the same period.

There were similar trends using time spent travelling as a denominator, with a 5% (IRR 1.052; 95% CI: 1.028–1.077, p < 0.001) annual increase in the pedestrian KSI rate per million hours use from collisions with pedal cycles, and an annual 2% reduction (IRR 0.975, 95% CI 0.972–0.978, p < 0.001) in pedestrians KSI from collisions with cars/vans during the same period. The results estimated by the Negative Binomial regression model were consistent with those from our Poisson model.

These trends were also robust to our sensitivity analyses. Trends were similar when using a different denominator (average distance travelled) and when using different numerators: KSI pedestrian injuries sustained single-vehicle collisions (with cyclists and motor vehicles), KSI pedestrian injuries sustained in all collisions with cyclists (including those with motor vehicles), and KSI pedestrian injuries sustained in all collisions with motor vehicles (including those with pedal cycles). Trends were also similar when data were excluded from police forces who adopted the new casualty severity recording system during our 2005–2015 study period.

## 4 Discussion

### 4.1 Implications of findings

Over the period 2005–2015 in England, there was an increase in cycling. The number of pedestrians KSI following a collision with a cycle within those years was about 1.3% of all pedestrians KSI on the road. The fatality figures (average three per year) are also much lower than pedestrian deaths from collisions with other vehicles. The burden of injury from pedestrian collisions with pedal cycles remained low during this period. However, the incidence of pedestrian casualties following collisions with cycles increased over the 11 years studied. This appears to be more than proportionate to the increase of cycle traffic during this period, measured by either miles or hours cycled. Therefore, this increase was unlikely to be due to an increase in pedal cycle traffic alone. Comparing the trends for each exposure over the study period suggests that the rate for pedestrians KSI in collisions with pedal cycles is increasing over the period whereas it is decreasing for those with motor vehicles. Overall, there is evidence that the rate of pedestrians KSI by collision with a cycle increased by 4% annually in England between 2005 and 2015.

Of particular concern is the older profile of those pedestrians KSI in collisions with cycles in England, with 42% over 60 years old, compared with the age profile of those KSI in collisions with motor vehicles. The fatality rate for this age group was also high. In this setting, this is unlikely to reflect greater pedestrian exposure, given that those over 65 are under-represented in those making walking trips in England (Department for Transport, 2019b). Whilst the cognitive declines of age and slower walking speeds may play a role, it is unlikely that these are a greater factor for collisions with pedal cycles compared with those with motor-vehicles.

One possibility is that bicycles are harder to see and hear, and they are may be increasingly ridden in pedestrian-congested areas where road infrastructure interventions have made cycling safer. Interventions that reduce average traffic speeds may have improved pedestrian and cycling safety in general (Grundy et al., 2009), but may also have had differential impact on cycle-pedestrian collision rates if they facilitate faster cycling speeds, less predictable cycling pathways (e.g. emerging from between slow-moving traffic lanes) or increased pedestrian exposure. Assessing the impact of new road infrastructure on overall and relative injury rates is complex. A meta-analysis of ‘safety in numbers’ studies found good evidence for increases in safety (particularly for pedestrians) when the proportion of pedestrians or cyclists in a locality rises (Elvik and Goel, 2019), but that too few studies had included good measures of infrastructure to assess the role of infrastructure in this effect. This analysis did not include injuries resulting from collisions between pedestrians and pedal cyclists. There remains limited evidence on how changing road infrastructures, particularly those implemented to increase cyclists' safety, impact on pedestrian safety. One observational study found cycling infrastructure to be a factor that contributed to older pedestrians being less able to cross roads within times allowed by signalised pedestrian crossings (Lachapelle and Cloutier, 2017). Detailed ethnographic studies on the complex interactions needed between different mode users in shared space to keep safe (Brown, 2012; Lloyd, 2019) also suggest that recent changes in this infrastructure may have changed the relative risks to pedestrians from cyclists and motor vehicles.

These interactions (such as glances, acknowledgements, small adjustments of speed or direction) are subtle, and become habituated over time: they are what enable people's taken for granted ability to navigate crowded and mixed-mode urban streets with safety and speed, most of the time. However, these tacit skills may be disrupted by infrastructure changes, as pedestrians and other road users learn to react to new road layouts, and changing traffic flows and speeds. The increase between 2005 and 2015 in pedestrian collisions with pedal cycles might therefore represent a relatively short-term reaction to changing road environments.

To test this speculation that effects might be short term, future research could usefully compare the changes we observed over an 11-year period to the period afterwards, from 2015 to 2020. From March 2020, the COVID-19 pandemic resulted in a series of short term and rapid changes in traffic volumes and mode share in Great Britain. Initial analysis suggests the pandemic has been associated with declines in road casualties for all transport modes (except pedal cyclists for some months) that were steeper than might have been expected from the decline in vehicle miles (Department for Transport 2021). In England, local authorities were encourage to invest in support and infrastructure to increase active travel (Department for Transport 2020), and there is evidence of significant proportions of commuters intending to change transport mode as a result of the pandemic (Harrington and Hadjiconstantinou 2022). Untangling the complex mix of factors that will impact on the relative increases or decreases in pedestrians injured in collisions with pedal cycles in recent years will, then, be challenging given the large changes in miles travelled, mode share and road layouts in many areas, and the possible changes in experience and expertise in road users as people adopt less habituated travel practices.

### 4.2 Comparison with other studies

Our finding of an increased collision rate differs from the few available studies from other settings over similar periods. In the US, Tuckel et al. (2014) suggested declining incidence of injuries to pedestrians from cyclists in New York and California between 2004 and 2011, and that males and children aged 0–15 were at higher risk. In Melbourne, Australia, O’Hern and Oxley (2019) did not identify a significant increase in cycle-pedestrian collisions resulting in a hospital emergency department visit or admission, or reported to the police, over the years 2006–2016, despite the rising popularity of cycling in the city. The increase we identified in England over a similar period may reflect different patterns of road use and mode share, but also a possible methodological difference — both Tuckel et al. (2014) and O’Hern and Oxley (2019) used population measures to estimate rates of injury, whereas we used distance and hours travelled. In Great Britain, Scholes et al. (2018) compared fatality rates for driving and cycling over a similar period (2005–2013), identifying higher fatality rates for driving, per hour travel time. As they note, although death rates for those in charge of a vehicle are higher for cyclists, once ‘third parties’ deaths are included, driving incurs a higher burden of fatalities. However, even pooling three years' worth of data, their study included too few deaths from collisions with cycles to make precise estimates of changing rates of injury.

### 4.3 Implications for policy and practice

Although increasing active travel has multiple benefits for public health, it is important to assess and manage potential risks as well. As cities across the world invest greater resources in cycling infrastructure, more evidence is needed on the impacts on pedestrian as well as cycling safety. A Cochrane review of the impact of cycling infrastructure on cyclists’ safety (Mulvaney et al., 2015) found insufficient robust evidence to make recommendations. There is, to date, even less evidence on the impact of cycling infrastructure on pedestrian safety. Pedal cyclists and pedestrians are both vulnerable road users – but road infrastructure changes, traffic mode mix, and other changes may have different effects on the two road user groups. That greater investment in safe infrastructure to accommodate both pedestrian travel and cycling is an urgent need is suggested by our findings that one in four KSI casualties occurred when the pedestrian was on a footpath or verge, and that a similar proportion were on pedestrian crossings. Few of these collisions were on space designed to be shared by pedestrians and pedal cycles, suggesting a certain amount of riding bicycles on footpaths, which is illegal in the UK. Survey evidence from other settings suggests that cyclists are more likely to break rules when existing infrastructure does not provide safe cycling routes (Shaw et al., 2014).

### 4.4 Strengths and limitations

The strengths of this study are that we used both cycle mileage and time cycled to assess trends over time, offsetting the weaknesses of each measure of exposure; we used a dataset which includes place of collision, providing essential information for policy and planning; and we used the most comprehensive data set on injuries resulting from collisions in Great Britain. There are, however, some limitations with using STATS19 data, including potential under-reporting of road traffic collisions (Department for Transport, 2013), changing reporting practices over time (Lyons et al., 2008) and exclusion of collisions that are not on the public highway, such as those in car parks, private driveways or off-road (Department for Transport, 2013). As STATS19 data only include injuries arising from collisions with vehicles, they also do not include injuries sustained, for instance, through trips or falls over road infrastructures, which may form a significant proportion of pedestrian injury on the highway (Methorst et al., 2017).

We focused on a period which saw increased cycle mode share, but were unable to assess the longer-term impacts of this change after 2015 due to the changing methods of identifying severity, which increased the number of causalities identified as ‘serious’ by police forces after 2015 (Department for Transport, 2018). We restricted our analysis to the years before this change was implemented, although pilot schemes were used by some police forces before then (Department for Transport, 2018). However, our sensitivity analysis suggested that removing data from these forces had no effect on trends identified.

There are limitations in both the denominator measures used. Vehicle miles are derived from manual counts and automatic traffic counters on major and minor roads, and data on road lengths (Department for Transport, 2016b), with milage calculated using growth factors and expansion factors and applied to the roads that were not counted. For cycling, the mileage data only provides estimates on public roads, cycle paths and footpaths adjacent to them, excluding cycle activity elsewhere, such as canal towpaths and bridleways (Department for Transport, 2017b). As collision data also only include collisions on public roads, this should not have resulted in cycle-pedestrian collisions per million miles cycled to be overestimated in this study. We may, however, have underestimated the increase in cycling (and thus overestimated the rate of injury per billion miles cycled) if mileage estimates do not adequately account for changing cycle routes as cycling increases. The other measure, estimated average time per mode, is unaffected by differential undercounting of traffic volumes, but may be vulnerable to reporting biases if, for instance, there have been changes in likelihood of reporting cycling or driving time over the years 2005–2015. Finally, we did not account for any temporal trends in pedestrian activity: both miles walked and number of walking trips began increasing in 2014, after declining for the previous 10 years (Department for Transport, 2019b). There are no perfect denominators for assessing injury rates, and certainly none which would enable meaningful direct comparisons of injury rates by mode. However, our use of two measures, with broadly aligned findings, does provide some reassurance that the findings are unlikely to be artefactual.

## 5 Conclusions

Increasing the amount of active transport is vital for progress towards healthy transport systems, particularly in urban areas. However, this should not be at the expense of vulnerable road users, particularly older pedestrians. We found that the incidence of pedestrians injured in collisions with pedal cycles increased in England over an 11-year period, with the growth rate faster than estimates of increase in cycle traffic. Further research is urgently needed on whether these are short-term impacts, and on what infrastructures best protect pedestrians, as well as cyclists, from the risks of road injury.

## Author statement

Tika Ram: Conceptualisation, data preparation & analysis, data interpretation, drafting and revising.

Judith Green: Conceptualisation, data interpretation, drafting and revising.

Rebecca Steinbach: Conceptualisation, data preparation & analysis, data interpretation, drafting and revising.

Phil Edwards: Conceptualisation, data preparation & analysis, data interpretation, drafting and revising.

## Financial disclosure

The authors have no financial conflicts of interest to disclose.

## Declaration of competing interest

The authors declare they have no competing interests.
