# Supplementary material for: Pedestrian injuries in collisions with pedal cycles in the context of increased active travel: Trends in England, 2005–2015
Source: J Transp Health. 2022 Mar;24:101340. doi: 10.1016/j.jth.2022.101340 (PMC8924875; doi:10.1016/j.jth.2022.101340)
Supplement: Multimedia component 1 [file mmc1.docx]

| **Casualty Severity** | **Pedestrian injured in collisions with one or more pedal cycles** | | **Pedestrian injured in collisions with one or more motor vehicles** | |
| --- | --- | --- | --- | --- |
|  | Number | % | Number | % |
| **Fatal** | 30 | 0.88% | 4,736 | 1.83% |
| **Serious** | 733 | 21.47% | 52,858 | 20.44% |
| **Slight** | 2,651 | 77.65% | 200,999 | 77.73% |
| **Total** | **3,414** |  | **258,593** |  |

Appendix, Table A.1:

Total number of pedestrians injured in collisions with pedal cycles and motor vehicles (2005-15)

Appendix, Table A.2:

Pedestrian KSI rates per billion vehicle miles (BVM) in Great Britain (2005-2015)

| **Year** | **Pedal Cycle** | | | | **Motor Vehicle^2^** | | | |
| --- | --- | --- | --- | --- | --- | --- | --- | --- |
|  | **KSI** | **Traffic Volume^1^** | **Rate** | **95% CI^3^** | **KSI** | **Traffic Volume^1^** | **Rate** | **95% CI^3^** |
| 2005 | 59 | 2.45 | 24.08 | 18.33 - 31.06 | 6,060 | 209.3 | 28.95 | 28.23 - 29.69 |
| 2006 | 43 | 2.55 | 16.86 | 12.20 - 22.71 | 5,986 | 211.2 | 28.34 | 27.63 - 29.07 |
| 2007 | 42 | 2.32 | 18.10 | 13.05 - 24.47 | 5,952 | 212.9 | 27.96 | 27.25 - 28.68 |
| 2008 | 49 | 2.58 | 18.99 | 14.05 - 25.11 | 5,638 | 210.4 | 26.80 | 26.10 - 27.51 |
| 2009 | 62 | 2.70 | 22.96 | 17.61 - 29.44 | 5,160 | 208.1 | 24.80 | 24.12 - 25.48 |
| 2010 | 74 | 2.72 | 27.21 | 21.36 - 34.15 | 4,813 | 204.6 | 23.52 | 22.86 - 24.20 |
| 2011 | 82 | 2.78 | 29.50 | 23.46 - 36.61 | 4,996 | 204.6 | 24.42 | 23.75 - 25.11 |
| 2012 | 79 | 2.82 | 28.01 | 22.18 - 34.91 | 5,169 | 203.2 | 25.44 | 24.75 - 26.14 |
| 2013 | 91 | 2.82 | 32.27 | 25.98 - 39.62 | 4,589 | 203.2 | 22.58 | 21.93 - 23.25 |
| 2014 | 94 | 3.11 | 30.23 | 24.42 - 36.99 | 4,668 | 208.7 | 22.37 | 21.73 - 23.02 |
| 2015 | 88 | 2.92 | 30.14 | 24.17 - 37.13 | 4,563 | 211.5 | 21.57 | 20.95 - 22.21 |
| Average | 69 | 2.71 | 25.63 | 23.84 – 27.51 | 5236 | 208.0 | 25.18 | 24.97-25.38 |

Notes

^1^. Billion Vehicle Miles; ^2^. Excluding motorway miles and motorway KSI; ^3^. Using Poisson Exact
